# A localization method for wireless capsule endoscopy using side wall cameras and IMU sensor

**DOI:** 10.1038/s41598-021-90523-w

**Published:** 2021-05-27

**Authors:** Seyed Shahim Vedaei, Khan A. Wahid

**Affiliations:** Department of Electrical and Computer Engineering, University of Saskatchewan, Saskatoon, SK S7N 5A9 Canada

**Keywords:** Gastroenterology, Engineering

## Abstract

Localizing the endoscopy capsule inside gastrointestinal (GI) system provides key information which leads to GI abnormality tracking and precision medical delivery. In this paper, we have proposed a new method to localize the capsule inside human GI track. We propose to equip the capsule with four side wall cameras and an Inertial Measurement Unit (IMU), that consists of 9 Degree-Of-Freedom (DOF) including a gyroscope, an accelerometer and a magnetometer to monitor the capsule’s orientation and direction of travel. The low resolution mono-chromatic cameras, installed along the wide wall, are responsible to measure the actual capsule movement, not the involuntary motion of the small intestine. Finally, a fusion algorithm is used to combine all data to derive the traveled path and plot the trajectory. Compared to other methods, the presented system is resistive to surrounding conditions, such as GI nonhomogeneous structure and involuntary small bowel movements. In addition, it does not require external antenna or arrays. Therefore, GI tracking can be achieved without disturbing patients’ daily activities.

## Introduction

Invention of wireless capsule endoscopy (WCE) was a breakthrough in diagnosing gastrointestinal (GI) problems. WCE is designed in a small size electronic device that the patient can swallow, and it then travels through the GI tract. A camera is placed on top of the capsule^1^, and continuously captures images and transmits them to a data logger outside of the body. Several severe diseases, such as obscure GI bleeding, ulcer infections, Crohn’s disease, tumor, celiac disease, Barrett’s esophagus, and cancer occur in different regions of the GI tract without any symptoms^2^. Available WCE devices either offer real-time video data or recording pH or gas profiles of the tract. Physicians then examine this information to detect any abnormalities. In another type of WCE, camera is replaced with sensors such as temperature, pressure, Potential of Hydrogen (pH)^3^ or light spectrum analyser sensors^4,5^. WCE could pinpoint the abnormalities in GI system. Image processing algorithms detect diseases and notify the doctors for further examination^6^. However, doctors are interested in images which are tagged by their location with respect to the GI pathway, so that they could examine the disease progression and treat accordingly. At the time of surgery, location of the abnormality helps doctors to correctly operate, or during the drug delivery, pharmacist can select the correct capsule and drug to deliver to the correct part of the body and activate at the point of abnormality^7,8^.

The topic of localizing the capsule inside GI has gained a lot of interests among researchers. Many attempts have been done till now. In a broader view, the position could be defined with respect to either GI track anatomy, or external reference point, such as an antenna. In most cases, electronic capsules equipped with transmitting modules which transfer data through Radio frequency (RF) signals. Hence, RF localization techniques are promising options. Nafchi et al.^9^ have utilized Directional of Arrival (DoA) and Time of Arrival (ToA) techniques in conjunction with Inertial Measurement Unit (IMU) sensor to locate the capsule. An arrangement of circular antennas array around the body receives the signals and measures the distance. Simultaneously, they are able to estimate the position and velocity of the capsule with the help of the IMU sensor. Non-homogenous environment inside the body leads to fluctuation of received signals, so they have used an extended Kalman filter to moderate the instabilities of signals. Their proposed method results up to 10 mm error in tracking the capsule position. Ting-Goh^10^ presents an integrated tracking method of DoA and IMU measurements. A combination of eight columns of array antennas outside of the body capture signals and measure the distance toward the capsule. In addition, an IMU sensor determine the heading direction, then Unscented Kalman Filter fuses all data to provide the capsule’s location in Three Dimensional (3D) space. Some articles used the Received Signal Strength Indicator (RSSI)^11–13^ to measure the signal level and anatomize the body with pre-defined propagation properties. Later, they extracted ^11^ the 3D position with average and maximum positional error of 37.7 mm and 114 mm, respectively. The accuracy is not enough to be considered as a general solution; technical problem such as line-of-sight put these methods into a challenge. On top of that, in practical experiments, the antennas are mounted on patient’s body which used as a reference for localization, while the reference body is also in motion.

Some works have focused on other external localization techniques based on magnetic field. The idea is to integrate a small magnet inside the capsule, then an external magnetometer will measure the magnetic field^14^; finally, methods such as triangulation locate the capsule in 3D space. The main advantage of these approaches over other localization methodologies is that low-frequency magnetic signals can pass through the human tissues without degradation, and it is an advantage over RF approaches which are depending on the RF signal’s strength. Another advantage is that, the magnetic sensors do not need the line-of-sight vision to detect the capsule. All in all, the computational complexity and overall accuracy of magnetic-based techniques are comparative to RF localization methods. Taddese et al.^15^ make use of magnetic-based localization on their paper and achieved position and orientation accuracies lower than 5 mm and 6°mm, respectively. A recent study by Shao et al.^16^, they have enclosed a magnet with bio-tissue and tried to localize it. They showed that the non-ferromagnetic bio-tissue has minor effect on the magnetic fields, so positioning accuracy will not be influenced by human tissues. In addition, their proposed method obtains the initial guess of position through the variance-based algorithm which leads to reduce the iterations and achieve higher localization accuracy. Shao reported the positioning error up to 10 mm and average orientation error of 12°mm. External positioning also is facing significant challenges, such as interference between multiple magnetic sources and uncertain initial guess for optimization algorithm which leads to drift in estimating the location. Moreover, the need for fixed position external reference is also limiting the application of magnetic localization methods for clinical and experimental usage.

Internal localization could be performed via image processing techniques. Lee et al.^17^ have equipped a traditional endoscopy system with an IMU sensor to get the camera’s heading in order to compensate the rotation. Lee’s system gives the rotation angles; however, the position is still unknown. Turan et al.^18^ have proposed a monocular visual odometry for WCE. Utilizing the advantage of deep Recurrent Convolutional Neural Networks (RCNNs), the system is trained in an end-to-end manner, so there is no need to fine-tune the network parameters. Similar to previous methods, image processing localization algorithms encounter with some critical issues, such as inappropriate accuracy due the lack of reference point inside the body. Considering occasional movements inside the body and limited number of frames captured and transmitted by the WCE, it will increase the positioning error.

Some other types of localization methods are also available, for example, Kalantar-Zadeh et al.^19^ developed an electronic capsule, capable of sensing various gases in the gut. This capsule can sense oxygen, hydrogen, and carbon dioxide. They performed a human pilot trial and sensed gas concentration profile with respect to GI organs. Their research showed that, carbon dioxide concentration profile is a promising option to localize the capsule inside the body. In a recent article by Jang et al.^20^, an endoscopy capsule has been developed with two cameras located at capsule’s sides and it paves the path for taking pictures of the internal GI’s walls. Their system has the ability to localize the capsule inside the GI by RSSI technique. They have claimed an average localization error of 1 cm.

Medical and radiological imaging, such as Magnetic Resonance Imaging (MRI), Computerized Tomography (CT), ultrasound, X-ray, and gamma ray techniques or hybrid methods are also considered for capsule localization. But they are not easily combined with WCE because of the necessity for continuous imaging over all examination process which may long as 8 hours.

Till now several available WCE localization methods have been described. There are a number of factors that need to be considered while performing a comparative assessment among different technologies. The accuracy of the location is important, but not the only index. For example, although radiation-based methods such as MRI and X-ray offer a high level of accuracy, it is not feasible to perform those methods for long time duration, and not desirable for the risk of radiation exposure. Robustness is another factor that indicates whether the method is capable of tracking multiple motions including the most challenging involuntary motion of the small bowel. The intestine has inherent motion inside the body. Hence, the capsule’s location is recorded according to the current state of the intestine. After a while, it may take a new orientation. The new orientation of the small bowel and the position of the capsule should be considered at the time of surgery or drug delivery. Another factor like patient’s comfort is important as well. Methods that are intrusive and disturb a person’s daily activity are not suitable. The need for external reference point may require a box antenna surround the patient’s body and limit his/her activities. The availability or need of hospital facilities is another contributing factor. Some methods require technicians to constantly monitor the patient during examination. All in all, there are considerable tradeoffs between different techniques of WCE localization.

Above all, most localization techniques suffer from non-predictable involuntary movement of the small bowel itself, which disrupts the relative movement between the human body and the sensor array. For example, the body may move according to an external coordinates system, while the GI organ itself may move or rotate within the body coordinates in a different direction. As of today, no method is able to address this problem. In the next section, we propose a new approach for WCE localization that addresses this unique issue and results in a better accuracy.

## Methods

Human GI tract is a tubular pathway, using this feature we have developed a device for WCE localization. To shed some light on the method, we started this section by an example. Thinking of a Metro which is travelling in a tunnel, a man in the wagon has a compass in his hand and he is looking toward the tunnel’s wall. The compass gives the wagon’s heading direction, in addition, that person by looking toward the tunnel’s wall knows how far it has traveled. Similarly, we have proposed a system that is composed of an IMU sensors, side wall cameras, processing unit and transceiver unit. The orientation of the device comes from an IMU sensor and capsule’s displacement derives from side wall cameras. On top of that, a fusion algorithm is introduced to combine the orientation data and the displacement. Finally, the system is able to plot the traveled trajectory in a 3D space.

### Capsule prototype

**Figure 1:**
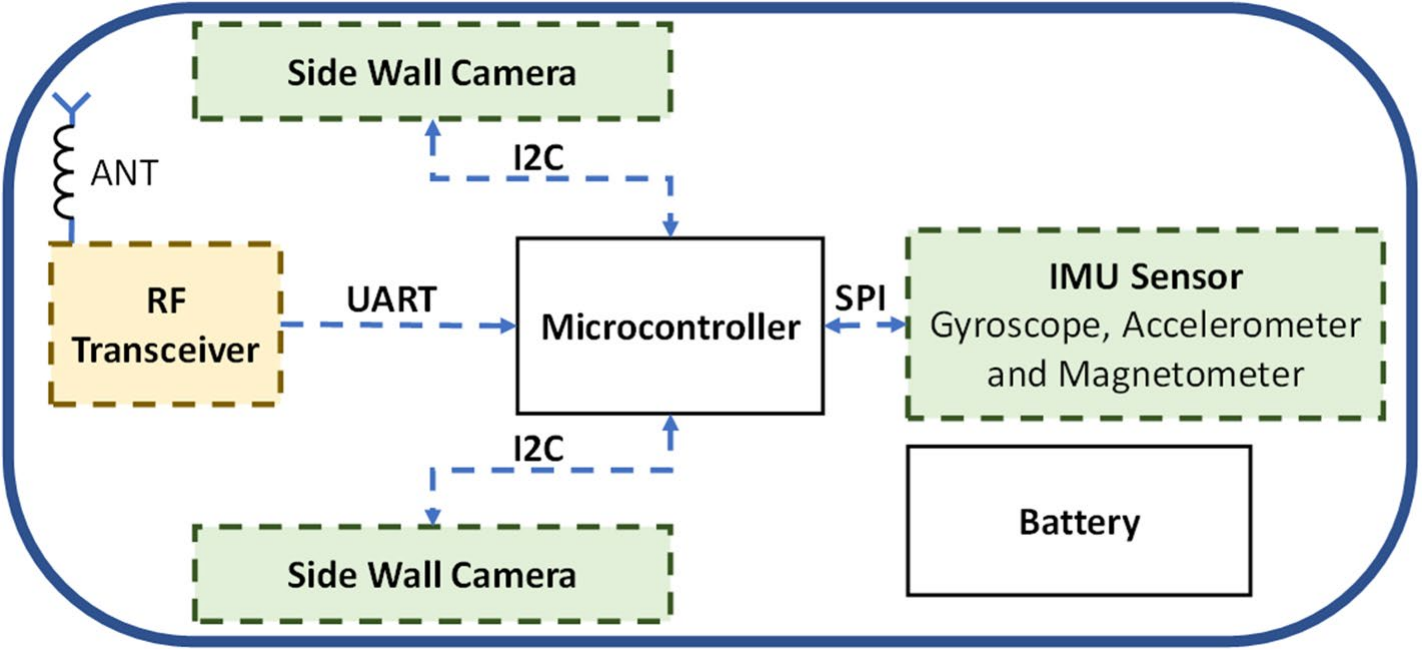
Schematic overview of the capsule.

**Figure 2:**
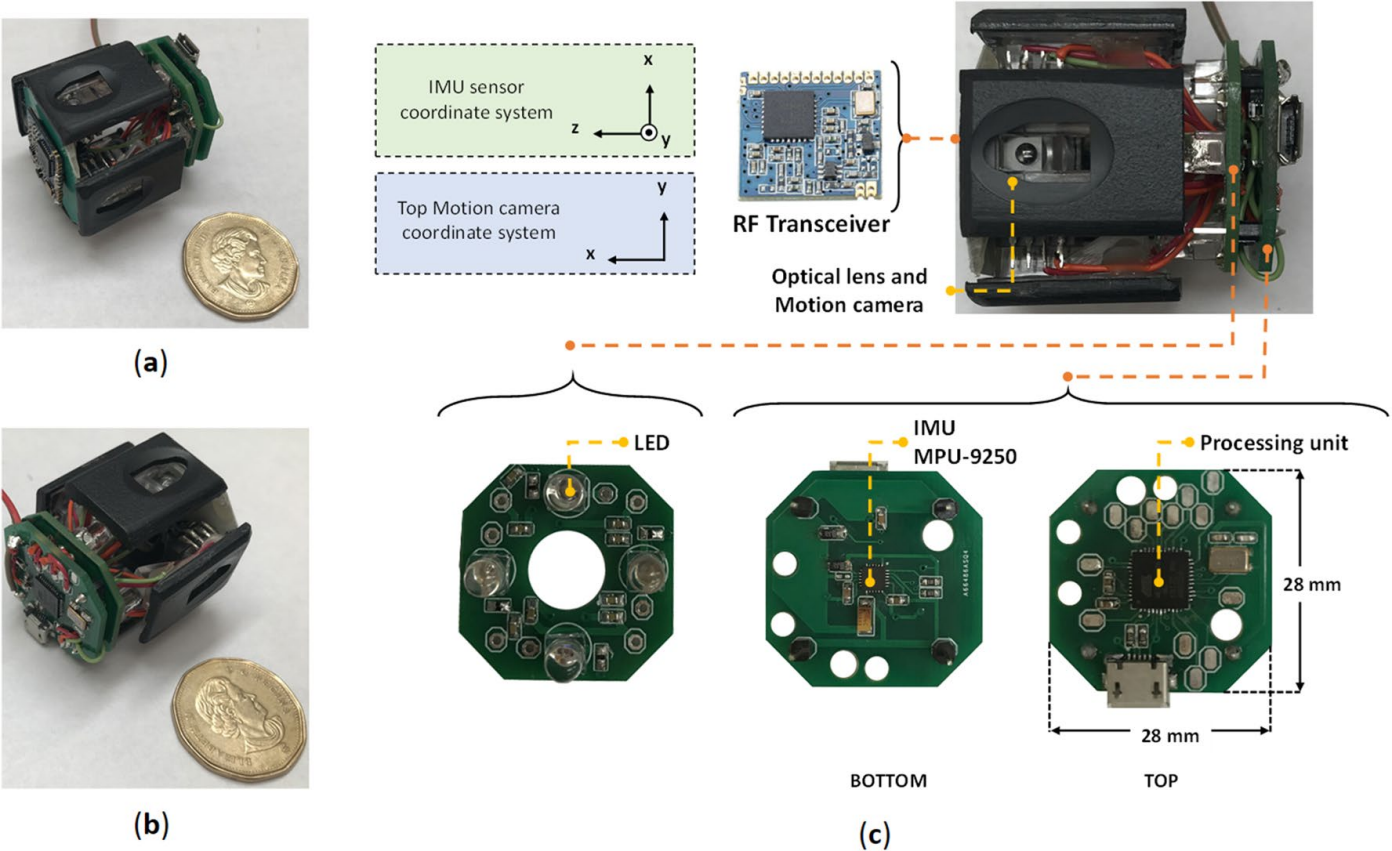
(**a**, **b**) Capsule prototype designed in the lab (**c**) Various parts of the lab prototype.

In this section an overview of the proposed device and necessary modules and components is provided. The system block diagram is shown on Fig. 1. The processing unit is a bridge between different parts of the system, and it controls all modules including IMU sensor, side wall cameras and RF transceiver unit. The capsule is equipped with four cameras, one for each side; however, to make it easy for presentation, only two of them is depicted in the schematic. The final prototype is shown in Fig. 2a,b. The capsule size is 3.5 cm × 3.5 cm × 4 cm. Different parts of the capsule prototype are shown in Fig. 2c. The capsule is responsible to capture the data and sends them in a raw format to the data-logger outside of the body; therefore, the capsule does not require any data processing which significantly reduces the design complexity. After receiving the data, a computer-based program is responsible to apply algorithms and calculate the 3D result.

### Processing unit

A microcontroller (µC) is responsible to connect all components together to work as a system. Individual sensors are connected to the µC through specific protocols. For this project we have used ATmega32U4 as a processing unit, this µC has built-in I^2^C and Serial Peripheral Interface (SPI) which make it simpler to use for different applications.

The µC reads all sensors then sends the raw data using a wireless link. The capsule reads 2 Bytes from each side wall camera. Considering four side wall cameras, the µC reads 8 Bytes in total. The IMU sensor produces 18 Bytes. Finally, the µC creates a frame of 28 Bytes with all data and necessary headers, then sends it to a data-logger outside of the body. In order to reduce the transmission power, the µC only sends the frame when it detects a motion.

### Side wall camera

Visual odometry is a technology that measures the displacement using optical flow of the scene. In principle, a camera captures the pictures and sends the frames out of the body for processing, then an image processing algorithm calculates the displacement. However, adding four cameras on capsule’s side and sending frames require huge power resources and transmission band width. While, processing images inside the capsule adds complexity to the design.

Motion measurement camera is an Integrated Circuit (IC) that has a low-resolution camera with on-board Digital Signal Processing (DSP) unit to run the image processing algorithms and communication interface, all on a small silicon dye.

**Figure 3:**
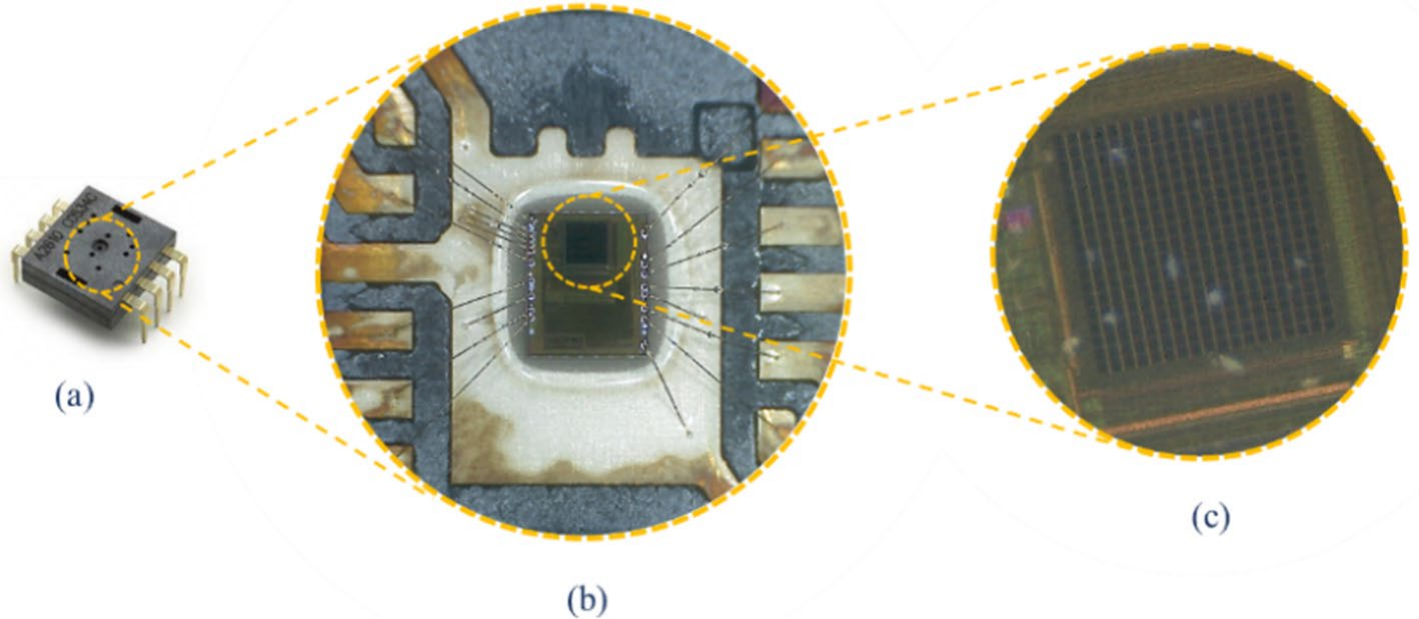
Optical motion measurement sensor (similar to the one used in computer optical mouse today), (**a**) dual inline package IC, (**b**) Microscopic view and (**c**) 18 × 18 pixels CMOS camera.

Figure 3 illustrates the side wall camera, the actual size of this IC is 1 mm × 2 mm. The light sensitive part is a Charged Coupled Device (CCD) camera that has 18 × 18 pixels sensor array. A chip with part number YS8008B is selected for the design. This IC works with 3 V power supply and it has Inter-Integrated Circuit (I^2^C) interface protocol.

**Figure 4:**
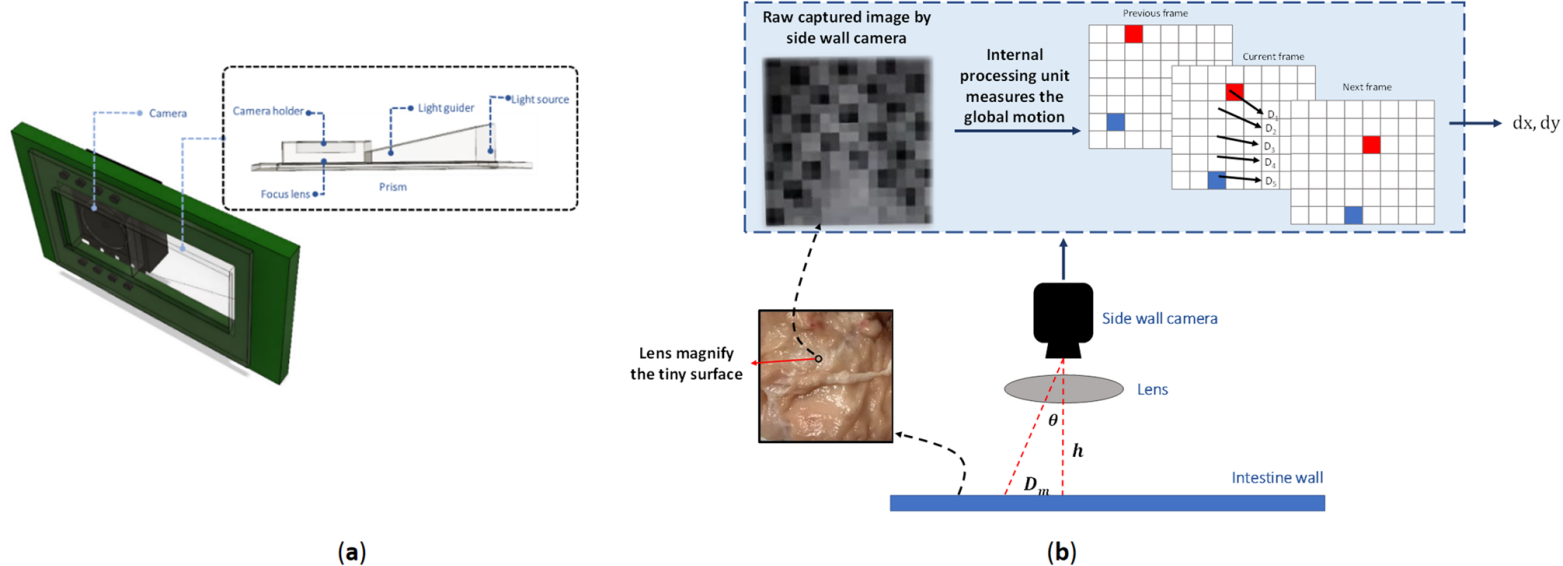
(**a**) Side wall camera and its optical parts, (**b**) global motion vector calculation.

The technology used inside wall camera is similar to the one used in optical mouse today. It consists of three basic components: motion sensor (monochromatic camera), optical lens and lighting source. Figure 4a illustrates the camera and related optical system. The optical system consists of two parts, a prism and a focus lens. The prism guides the light source so that it lights up the camera view then the lens focuses the reflected light from surface to the CCD. The light guider is positioned so that it could receive the light rays from the right side and guides the rays with a correct angle to be emitted to the surface and reflected precisely to the CCD. In addition, a Light Emitting Diode (LED) with red color is selected to have the highest reflection rate in the GI. A magnifier (lens) is placed between the side wall camera and surface. This lens magnifies the surface as shown on Fig. 4b and makes it possible to see several tiny markers. The raw image has only 18 × 18 pixels of a microscopic view of the surface. Hence, the motion sensing does not depend on larger markers on GI’s walls, instead it depends on global motion. The processor inside the side wall camera is responsible to measure the global motion vectors. The side wall camera takes pictures every 20 ms and by looking at global motion of pixels frame to frame, it can detect the size and direction of motions. A separate source of light is used for side wall camera to adjust the brightness. In addition, due to the configuration of side wall cameras around the capsule, at least one of them always is attached to the GI’s wall. By tuning the correct focal length (*h*), pixels are less likely to get blurred. However, in such cases, the entire figure also moves, and processor still is be able to derive the motions.

Figure 4b shows how the motion estimation algorithm is working. $h$ is the fixed distance between the camera and intestine’s wall. $\theta$ is the angular position of each pixel, and $\overrightarrow{D}$ is a vector that shows the displacement of individual pixels compared to the previous frame. The displacement comes from four cameras which are located at the capsule’s side wall. As a rule of thumb at least one camera should stick to the intestine’s wall. The camera takes pictures every 10 ms. Then, pictures are compared and distinct points at each frame will be selected. Then, the algorithm looks for the same point at adjacent frames. The algorithm is able to calculate motion vectors for individual pixels (${\overrightarrow{D}}_{1}, {\overrightarrow{D}}_{2} , \dots$). Finally, it computes the average vector and finds the average displacement vector ${(\overrightarrow{D}}_{m})$**.**

### Inertial measurement unit

An Inertial Measurement Unit, commonly known as an IMU, is an electronic device that measures and reports orientation, velocity, and gravitational forces using accelerometers and gyroscopes and often magnetometers. For this project we have used an IMU sensor MPU9250 with size of less than 5 mm × 5 mm.

**Figure 5:**
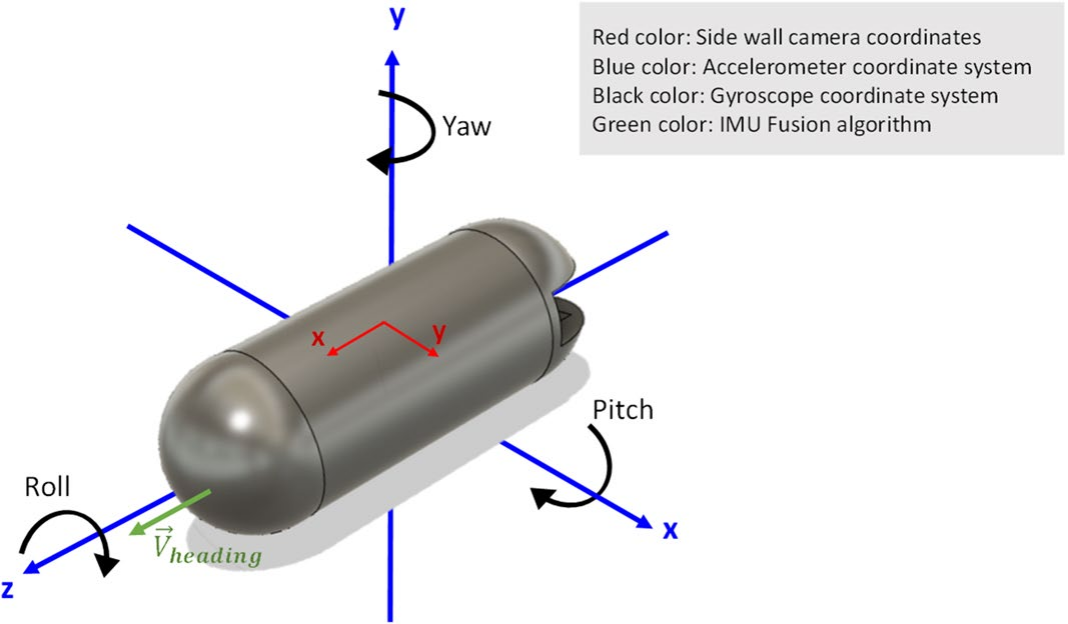
Rotation about different axes.

Gyroscope measures angular velocity, in other words gyroscope reports how fast the device is spinning about an axis. Rotation about different axes is illustrated in Fig. 5, which named as roll, pitch and yaw. The gyroscope gives the rate of change of the angular position over time with a unit of $\left(\frac{\mathrm{deg}}{\mathrm{s}}\right)$, according to Eq. (1).

1 $$\dot{\theta }=\frac{d\theta }{dt}$$

In order to derive angular position, we integrate the angular velocity in a period of time, it can be mathematically shown as Eq. (2)

2 $$\theta \left(t\right)={\int }_{0}^{t}\dot{\theta }\left(t\right)dt\approx {\sum }_{0}^{t}\dot{\theta }\left(t\right){T}_{s}$$

where, *T*_*s*_ is the sampling time and it represents the time interval between samples. The gyroscope data is reliable only on the short term, as it starts to drift on the long term. Hence, accelerometer data is used to compensate the gyroscope data.

The accelerometer measures all forces that are working on the object and sends them as ${A}_{x}$, ${A}_{y}$ and ${A}_{z}$. To obtain the angular position with the accelerometer, we are going to determine the position of the earth gravity vector which is always visible on the accelerometer. This can be done by using an atan function. In addition, the yaw derived from magnetometer; so, a fusing algorithm is required to combine the sensors all together.

IMU sensor and side wall cameras have different coordinate systems. The IMU’s z axis is placed along the front to back of the capsule as depicted on Fig. 5. The heading vector (${\overrightarrow{V}}_{heading}$) shows the capsule’s motion direction (IMU’s z axis) in a 3D space. ${\overrightarrow{V}}_{heading}$ is defined based on the earth gravity and earth north magnetic pole.

The IMU sensor must be calibrated before use to compensate the surrounding conditions and noises. In order to produce an accurate orientation estimation, the magnetometer should see the earth’s magnetic field, and any ignore conditions that affect the earth’s magnetic properties. The magnetic measurements will be subjected to two types of distortion—hard iron and soft iron distortions. Objects that produce a magnetic field cause the hard iron distortion, which is a permanent bias in magnetometer output. Soft iron distortion, on the other hand, caused by the ferromagnetic objects, and leads to deflections or alterations in the existing magnetic field. In order to cancel hard iron effect bias terms should be added to the raw data, meanwhile, correction factors in matrix format will multiply to the measured magnetic field to neutralize the soft iron distortion according to Eq. (3)

3 $$\left[\begin{array}{c}\begin{array}{c}{M}_{xc}\\ {M}_{yc}\end{array}\\ {M}_{zc}\end{array}\right]=\left[\begin{array}{ccc}{S}_{11}& {S}_{12}& {S}_{13}\\ {S}_{21}& {S}_{22}& {S}_{23}\\ {S}_{31}& {S}_{32}& {S}_{33}\end{array}\right]\times \left[\begin{array}{c}\begin{array}{c}{M}_{x}\\ {M}_{y}\end{array}\\ {M}_{z}\end{array}\right]+\left[\begin{array}{c}\begin{array}{c}{B}_{x}\\ {B}_{y}\end{array}\\ {B}_{z}\end{array}\right]$$

where, ${M}_{x}$, ${M}_{y}$ and ${M}_{z}$ are magnetic fields read from the sensor. ${M}_{xc}$, ${M}_{yc}$ and ${M}_{zc}$ are calibrated magnetic fields. ${B}_{x}$, ${B}_{y}$ and ${B}_{z}$ are the applied biases to compensate the hard iron effect, and ${S}_{mn}$ are correction factors to remove the soft iron distortion.

### Transceiver

At this stage of designing we are not concerned about modules’ size, so we have hired a Lora RF module operating at 433 MHz. The Lora is configured on Transmitter/Receiver (TX/RX) mode, which is perform as serial wireless bridge, the µC sends and receive data through Universal Asynchronous Receiver–Transmitter (UART) protocol connecting to TX/RX pins via Lora.

### Sensor fusion algorithm

**Figure 6:**
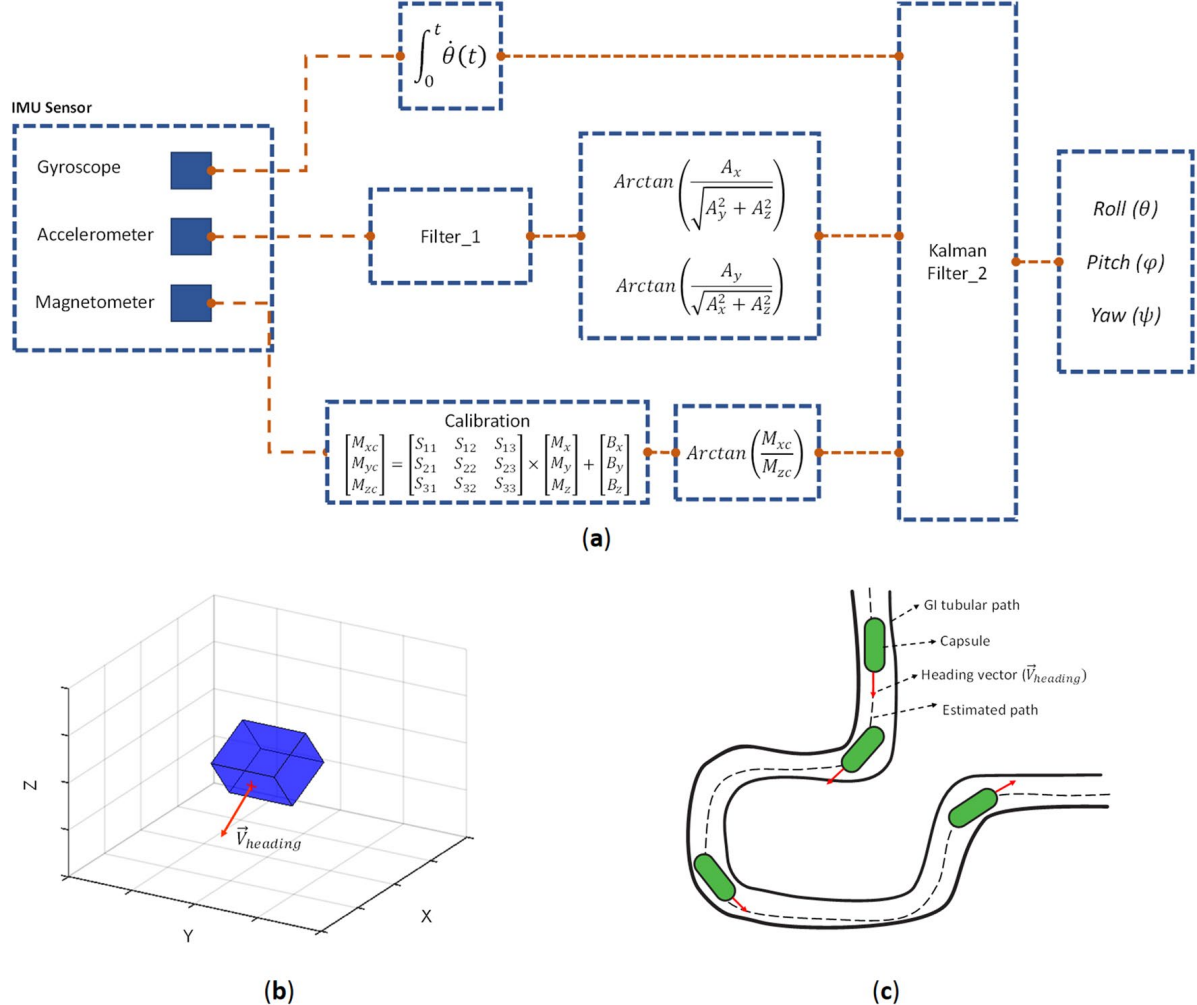
(**a**) IMU sensor fusing schematic, (**b**) 3D orientation result and (**c**) overview of the fusion algorithm.

Generally, in order to get 3D orientation of the capsule, we have to fuse all three sensors of accelerometer, gyroscope and magnetometer. Kalman filters have been demonstrating its usefulness in various applications, it not only works well in practice, but is theoretically attractive because it can be shown that of all possible filters, it is the one that minimizes the variance of the estimation error. Kalman filtering is an algorithm that provides estimates of some unknown variables given the measurements observed over time. Figure 6a demonstrates the IMU sensor fusing schematic as described by Roetenberg et al.^21^. The accelerometer is designed based on a MEMS technology and suffers from noises on its output. Hence, Filter_1 is used to filter the noise. However, Kalman Filter_2 is part of the fusion algorithm and fuses the IMU data to find the orientation of the capsule. Figure 6b shows the 3D orientation result. The output provides an accurate orientation estimation.

The next step toward capsule localization is fusing the orientation with displacement data. The capsule’s orientation is derived via IMU fusion algorithm; and the heading vector can be calculated using roll, pitch and yaw angles according to Eq. (4). The heading vector should be normalized for further processing. By moving along the heading vector, we could compute the location in 3D space

4 $${\overrightarrow{V}}_{heading}=\left(-\mathrm{cos}(\psi )\mathrm{sin}(\varphi )\mathrm{sin}(\theta )-\mathrm{sin}(\psi )\mathrm{cos}(\theta ) \right)\widehat{X}+\left(-\mathrm{sin}\left(\psi \right)\mathrm{sin}\left(\varphi \right)\mathrm{sin}\left(\theta \right)+\mathrm{cos}\left(\psi \right)\mathrm{cos}\left(\theta \right)\right)\widehat{Y}+\left(cos(\varphi )sin(\theta )\right)\widehat{Z}$$

where, $\psi$ is yaw, $\varphi$ pitch and $\theta$ roll angles. The $\widehat{X}, \widehat{Y}$ and $\widehat{Z}$ are the unit vectors in Cartesian coordinate system.

Side wall camera sensor provides the displacement along the heading vector, then multiplying the displacement to heading vector represents the 3D projection of the capsule’s movement. This is shown in Eq. (5)

5 $$\overrightarrow{D}={D}_{mx}.{\overrightarrow{V}}_{heading}$$

where, $\overrightarrow{D}$ shows the 3D displacement vector, and ${D}_{mx}$ is the $\widehat{X}$ component of the ${\overrightarrow{D}}_{m}$. Finally, to get the 3D position, we have used the following equation:

6 $${P}_{t+1}(x,y,z)={P}_{t}(x,y,z)+\overrightarrow{D}$$

where, ${P}_{t+1}$ is the new position and ${P}_{t}$ is the previous position. Thus, we are able to calculate the new position of the capsule from the previous position. Finally, a 3D trajectory is generated.

Since the capsule is confined in a tubular shape track, like GI tract, peristalsis motion pushes the capsule through the cylindric structure and it moves only along ${\overrightarrow{V}}_{heading}$ (IMU’s z axis, and not along x or y direction). Capsule can move either forward or backward (± IMU’s z axis) depending on how it is entered into the small bowel. As shown in Fig. 6b the red vector defines the ${\overrightarrow{V}}_{heading}$, which pointing out the direction of capsule. The algorithm is designed in a way that other motions except ${\overrightarrow{V}}_{heading}$ will be ignored, as a result, intestinal motions inside body will not interfere the capsule tracking. An overview of heading vector and fusion algorithm is depicted on Fig. 6c.

## Results and discussion

### Surface pattern

**Figure 7:**
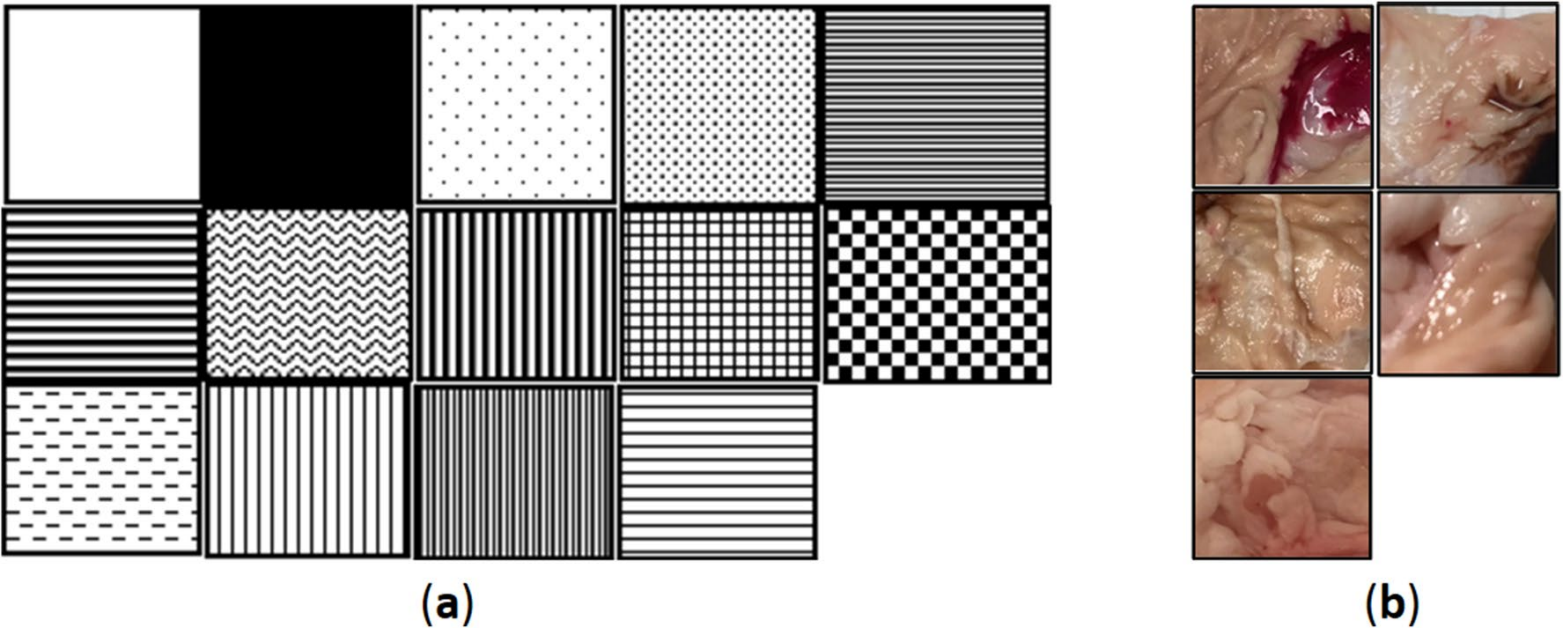
(**a**) Basic patterns, (**b**) WCE-like colored patterns.

In order to evaluate the motion sensor’s performance, basic patterns were introduced as depicted in Figure 7a to understand the preliminary movement of the device against surfaces with various patterns. Patterns are expanded on horizontal columns and printed on white paper. We placed the wall camera on top of that line and made multiple movements. The camera sensors were able to capture the dx displacement. The experiment has two outcomes. First of all, YS8008B could measure the displacement in all patterns except two of the parallel patterns. Secondly, the sensor’s sensitivity and lens’ focal length are important properties for correctly capturing and analyzing the surface. We expanded the experiments to WCE like patterns as shown in Fig. 7b. In this way, we make sure that wall camera will work on small intestine’s texture, as well.

The next objective is see if the camera can track the motion on a soft surface (like animal skin or meat). The surface’s material defines the optical reflection properties of the surface, several surface materials, such as porcine intestine, grounded meat and human skin, are selected. By considering a curved surface, side wall camera works as long as the top of the surface sticks to the motion sensor’s focal length. Elastic properties of the intestine will ensure that the surface is kept at the right distance from the lens. The initial experiment shows that the selected side wall camera is able to measure the traveled distance on soft surfaces like porcine intestine.

### Test setup

**Figure 8:**
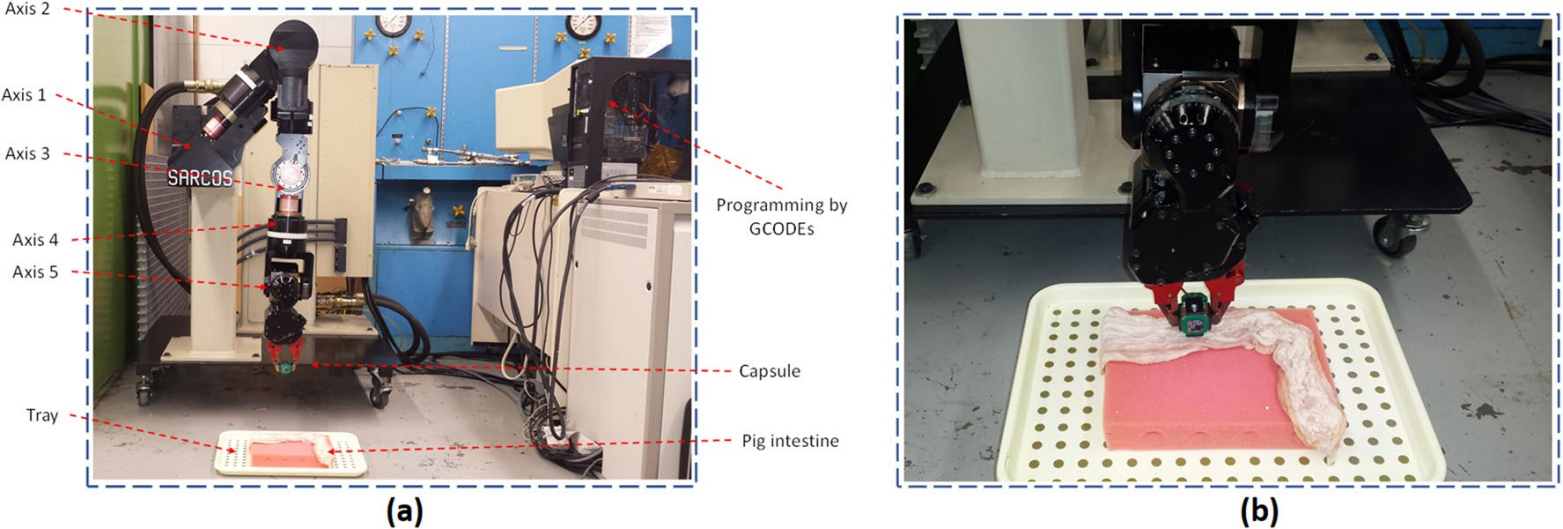
(**a**) Robotic arm experimental setup, (**b**) Capsule placement and porcine intestine.

In order to have a precise ground truth for verification, a robotic arm with five axis of freedom is used in all our experiments that holds the capsule prototype (Fig. 8a). The device sends raw data to a computer which then stored in an excel format for further processing.

The robotic arm is programmed using GCODEs to simulate capsule’s pre-defined motion inside the GI, including peristalsis and involuntary motion, and random motions across different trajectories. Hence, the ground truth is defined and can be used to compare with the estimated trajectory. The robotic arm has five axis of freedom which make it a suitable testbed for all experiments. Figure 8b illustrates a closer look at capsule and robotic arm.

The following types of motions were simulated using the setup to evaluate the performance of the proposed prototype and fusion method.

### 1D tracking

**Figure 9:**
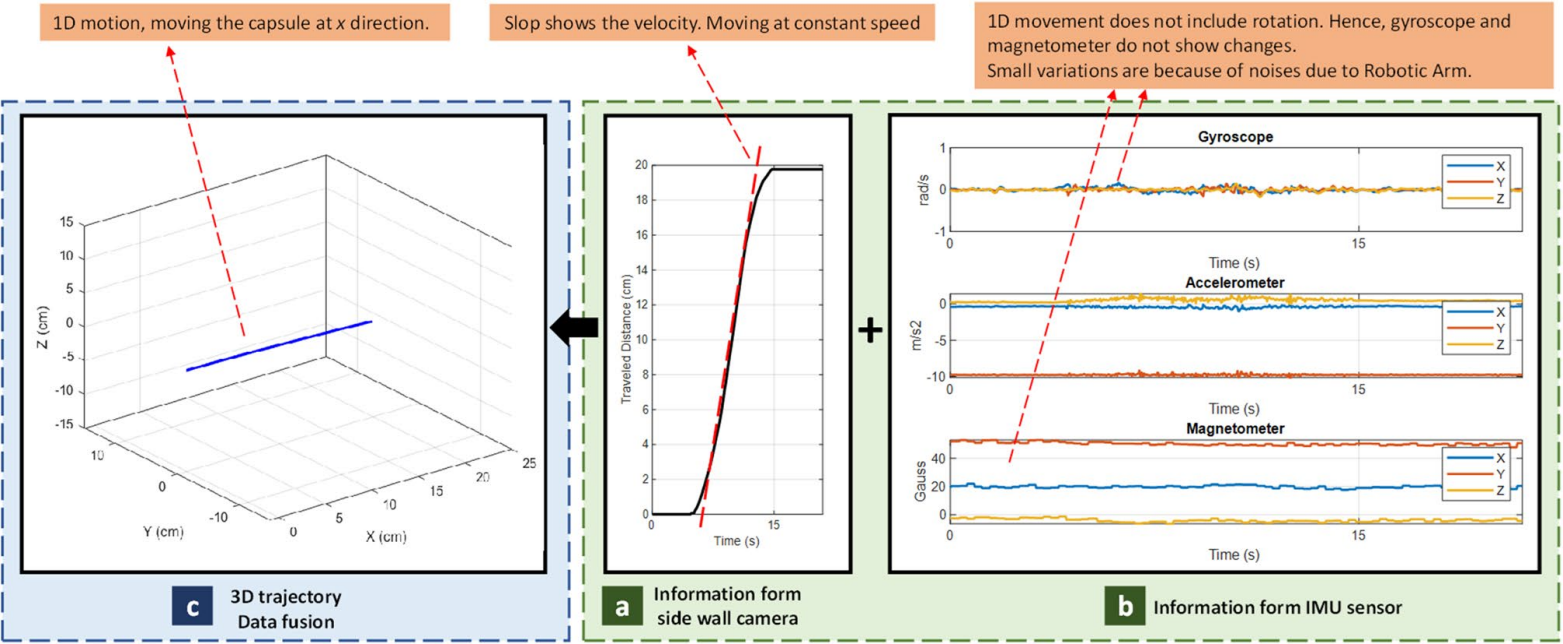
Results of 1D tracking.

This experiment is designed to evaluate the accuracy of the side wall camera for distance measurements. A robotic arm moves the capsule toward the *x* direction and side wall camera measures the distances. Figure 9 presents the 1D tracking data, in which Fig. 9a is the raw data from side wall camera. The slop of traveled distance plot indicates the velocity, Fig. 9b is the IMU data and Fig. 9c is the output of our fusion algorithm (estimated trajectory). Figure 9b shows minimal changes in Gyroscope and Magnetometer outputs which indicates that moving the capsule in a line does not include rotation.

**Figure 10:**
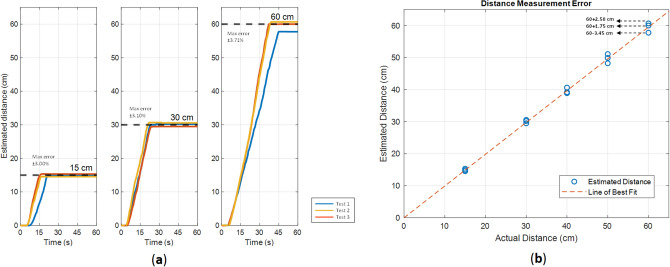
(**a**) Repeat the same path (**b**) Distance measurement error.

Figure 10 illustrates another experiment to evaluate the motion sensors’ accuracy. In this experiment, the capsule travels three straight line path with size of 15, 30 and 60 cm. Each experiment repeated three times. Figure 10a shows the experiment results and Fig. 10b illustrates the error in estimating the distance. It is shown that the maximum error of ± 3.71% can be achieved in 60 cm path.

### Leap and peristalsis motions

**Figure 11:**
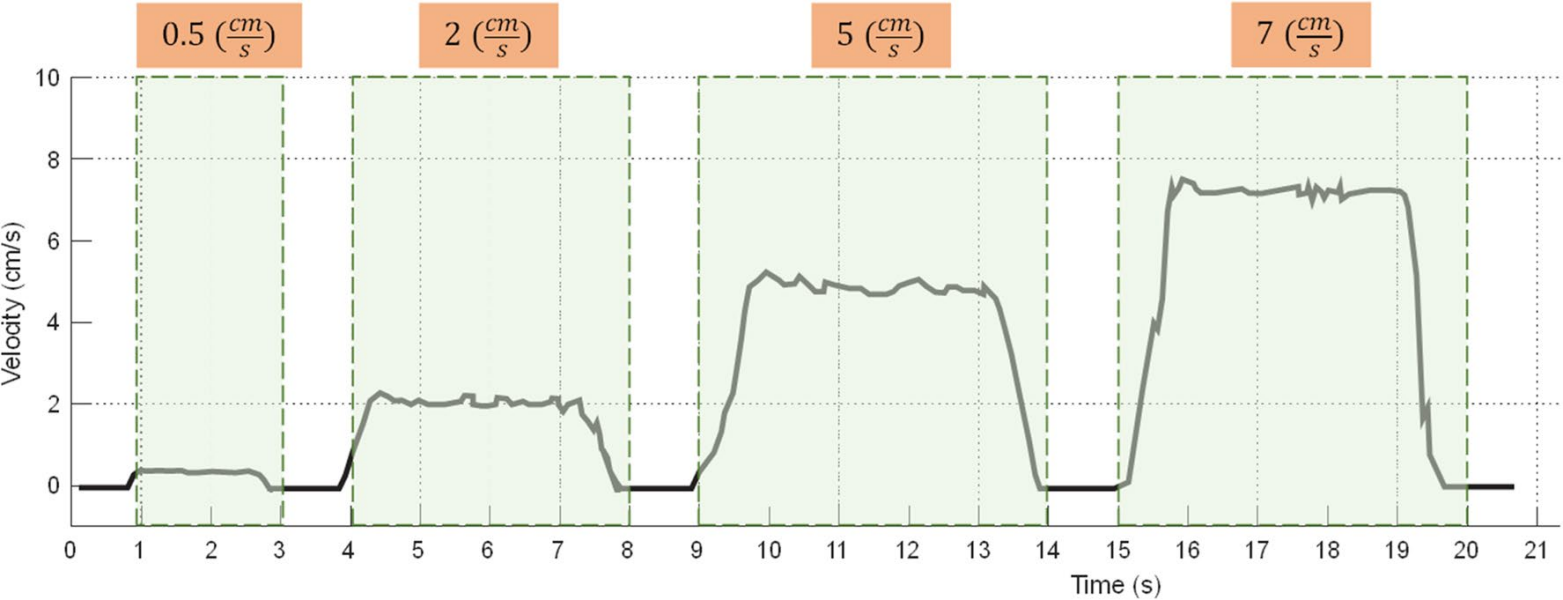
Results of Leap motion analysis.

Leap and peristalsis motions are two common motions inside the GI system. Leap motion consists of series of fast and small movements. The side wall camera measures these motions. Hence, the frame rate plays a key role. The YS8008B measures the displacement between two sampling time (T_s_) then sends the information to the host, hence, T_s_ limits the maximum speeds. Shorter T_s_ tends to overflow the buffer, so, the error rises in displacement measurement, on the other side, longer T_s_ results higher drift error. This experiment is performed to understand whether the side wall camera can detect surge motions. Four different speeds are evaluated including 0.5 cm/s, 2 cm/s, 5 cm/s, and 7 cm/s. As shown on Fig. 11, the side wall camera is able to successfully track velocity up to 7 cm/s. Note that the typical speed of capsule inside the small bowel is 2–5 cm/s^22^.

In another experiment, the peristalsis motion is investigated. Peristalsis motion is an involuntary contraction and relaxation of muscles which leads to push food ahead of the wave. Muscle’s contraction behind the food to keep it from moving backward, then longitudinal contraction to push it forward. Commonly, peristaltic waves exist in the small intestine at irregular intervals and travel for different distances, some ways travel only a few inches, others travel longer. Tracking an object which is under peristalsis motion could be difficult, due to the fact that the object might go forward and slightly backward.

**Figure 12:**
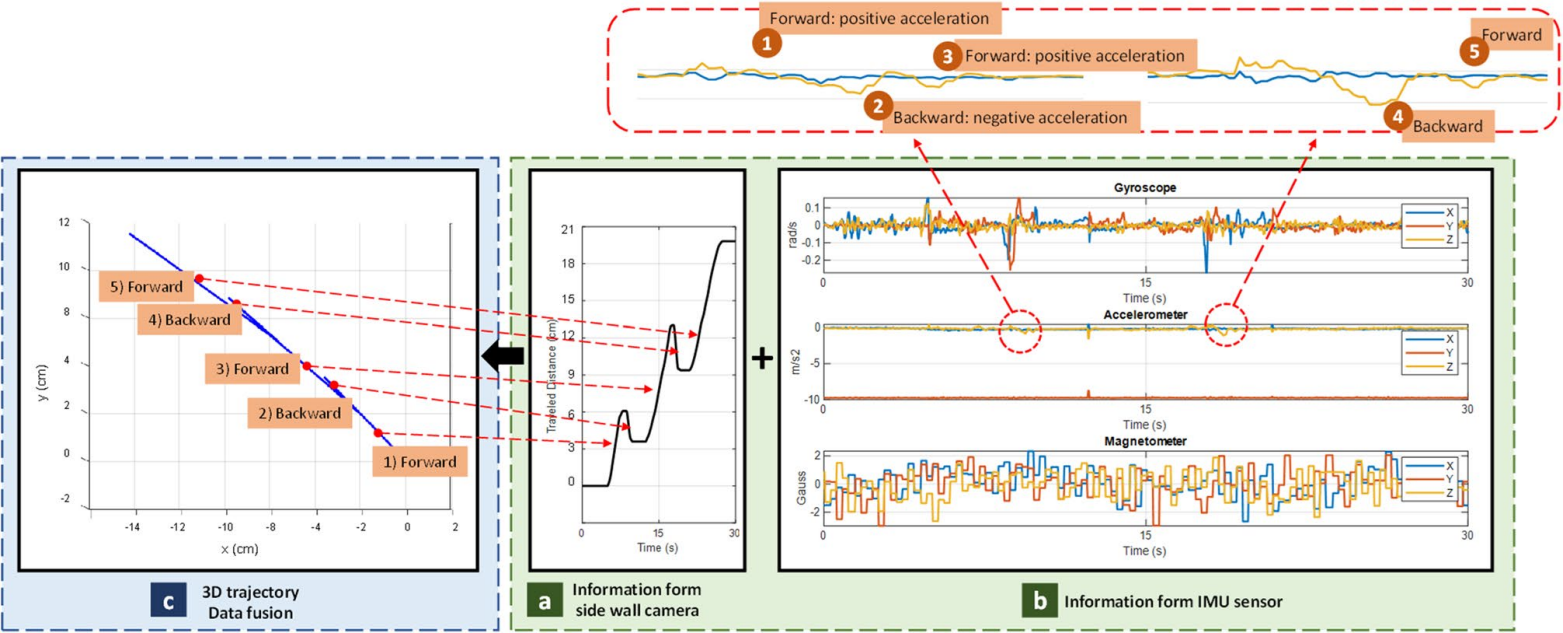
Results of Peristalsis motion analysis.

In this experiment, the robotic arm moves forward and backward in *x* direction to simulate the GI’s peristalsis motion. Figure 12 illustrates a peristalsis motion. In Fig. 12c, the 3D trajectory, fused by our system is shown for the forward and backward motions. The results show a relationship between the direction of travel and acceleration, as shown on Fig. 12b. The blue curve and yellow curve show accelerations across *x* and *z* axis, respectively. According to the plots, acceleration variation is reflected only on heading vector which is the IMU’s *z* axis.

The peristalsis motion’s sequence includes.

1. Forward motion
  i. constant speed (velocity: +, acceleration: 0)
2. Backward motion
  i. slow down until complete stop (velocity: +, acceleration: −)
  ii. speed up at opposite direction (velocity: −, acceleration: +)
3. Forward motion
  i. slow down until complete stop (velocity: −, acceleration: −)
  ii. speed up at opposite direction (velocity: +, acceleration: +)

This sequence is depicted at Fig. 12. The size of acceleration depends on the rate of velocity changes.

**Figure 13:**
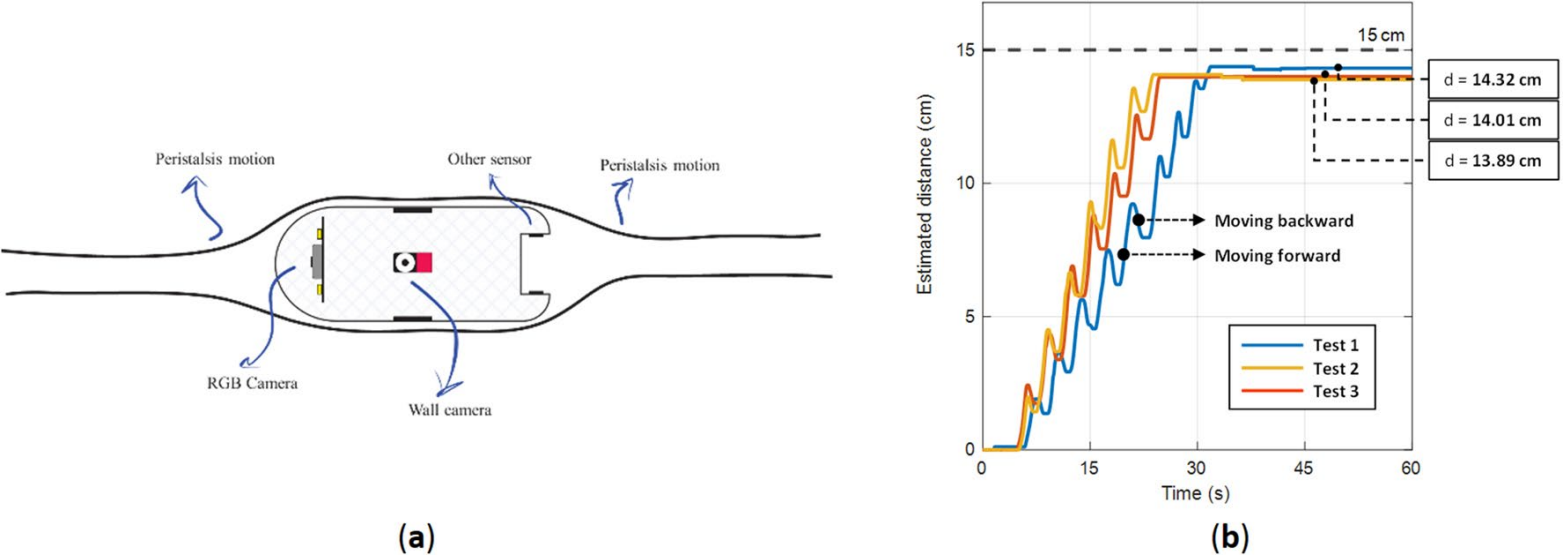
(**a**) Peristalsis motion (**b**) experiment results.

Figure 13a shows how peristalsis motion is working, due to the contraction of muscles, we could ensure that side wall cameras will stick to the GI wall. An experiment has performed in Two Dimensional (2D) tracking to monitor the side wall camera behaviors’ in such movement, as well. Figure 13b illustrates the effect of peristalsis motion in the experiment. The capsule is traveled a 15 cm path and during the path, it moves forward and backward several times. The results show that under this condition, the error in estimated distance is around 7.4%.

### Involuntary motion

Involuntary motion is the movement of small bowel inside the body coordinate. At this type of motion, capsule has a stationary location compared to the global GI system. However, its relative position within body coordinate will change. To simulate the involuntary motion, the capsule is attached to the intestine and placed on a tray, and a Robotic Arm moves the tray.

**Figure 14:**
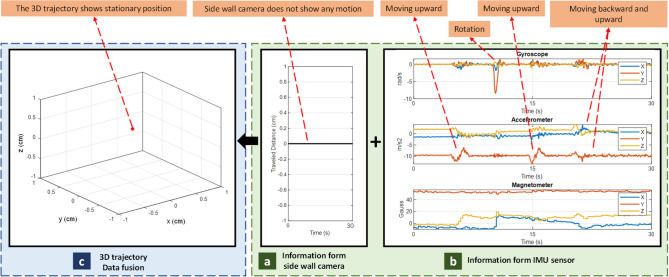
Results of Involuntary motion analysis.

During the involuntary motion, side wall camera will not detect any motion (Fig. 14a). Meanwhile, the simulated body movements are captured by the IMU sensor (Fig. 14b). Since, during involuntary motion, the capsule is stationary, but the small bowel is moving, the 3D trajectory is drawn as a single point showing no motion (Fig. 14c). Therefore, our method is able to detect the involuntary motion successfully.

### 3D tracking

**Figure 15:**
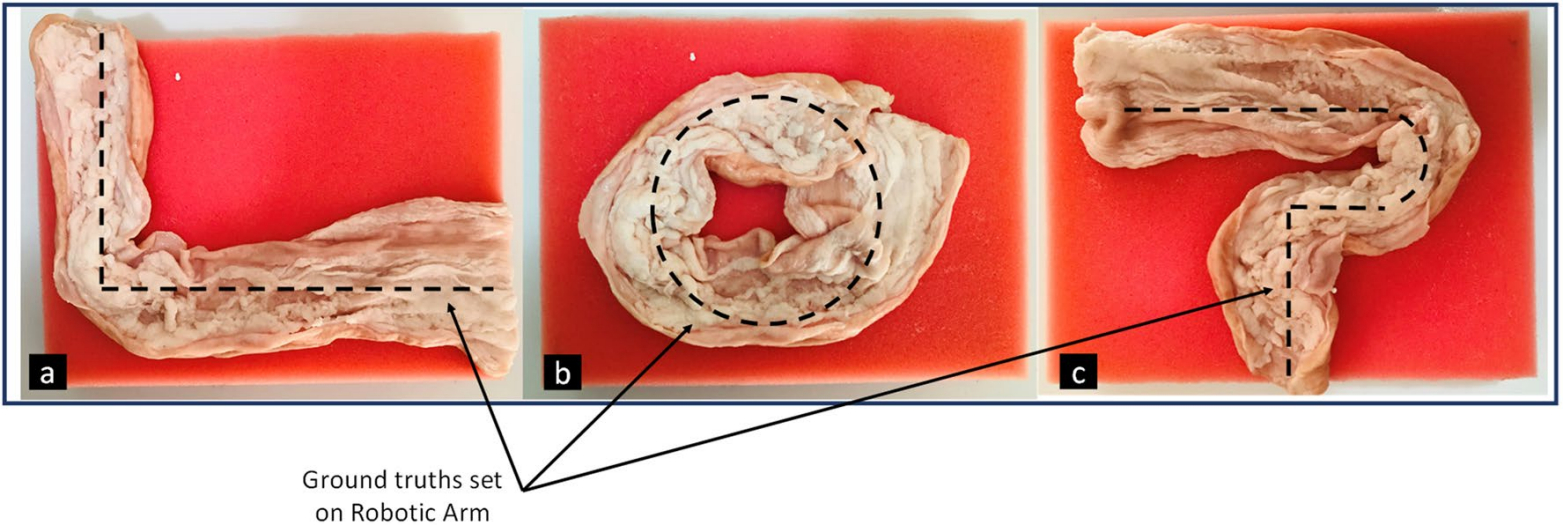
A piece of porcine intestine for test surface.

A robotic arm moves the capsule based on a predefined trajectory (ground truth). Several tracks are examined, but only three of them are shown on Fig. 15. Then, the proposed algorithm fuses information from side wall camera and IMU sensor to estimate the 3D trajectory.

**Figure 16:**
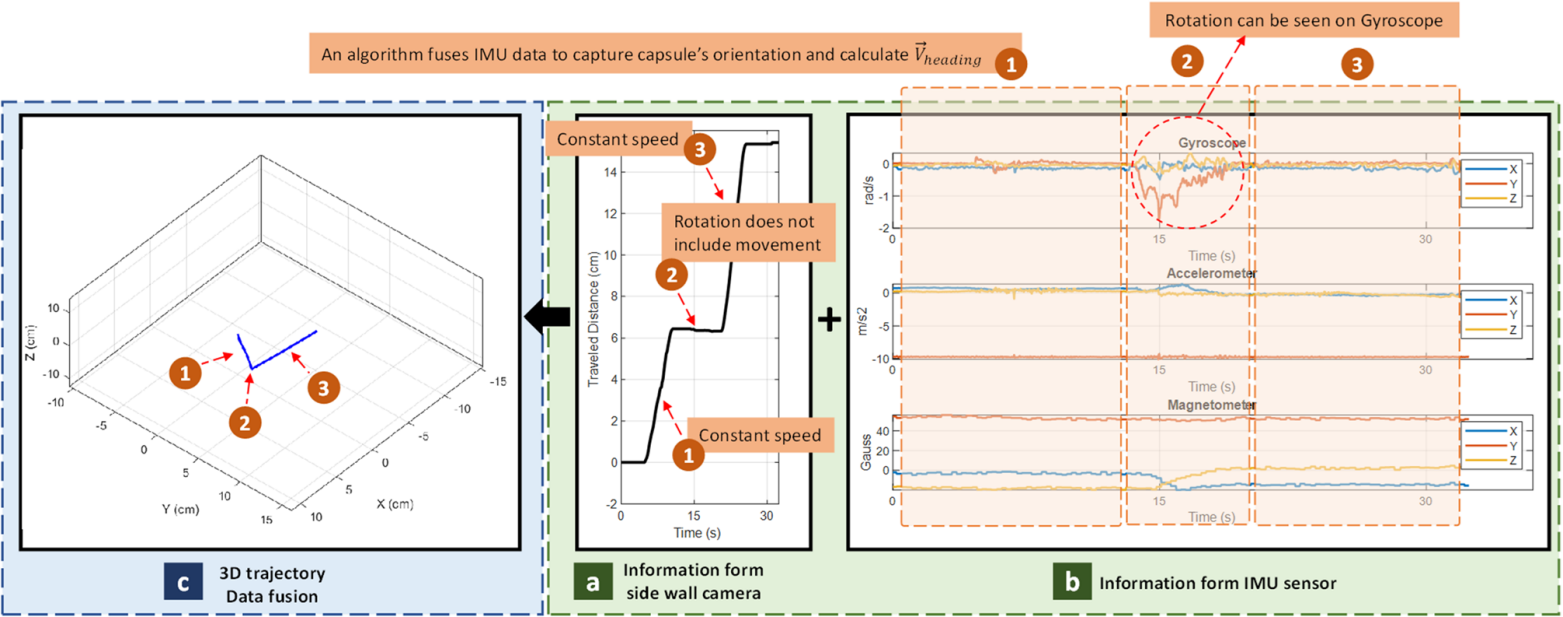
Results of 3D tracking analysis.

For the sake of explanation, the recorded data from trajectory of Fig. 15a will be described. As illustrated on Fig. 16, the trajectory is divided into three sections, (1) moving with constant speed at ${V}_{heading(1)}$, (2) 90° rotation, (3) moving with constant speed at ${V}_{heading(2)}$. As shown on Fig. 16a, during (1) and (3), side wall camera detects motions, but during (2) it does not show any motion, instead, IMU data shows rotation during this time.

**Figure 17:**
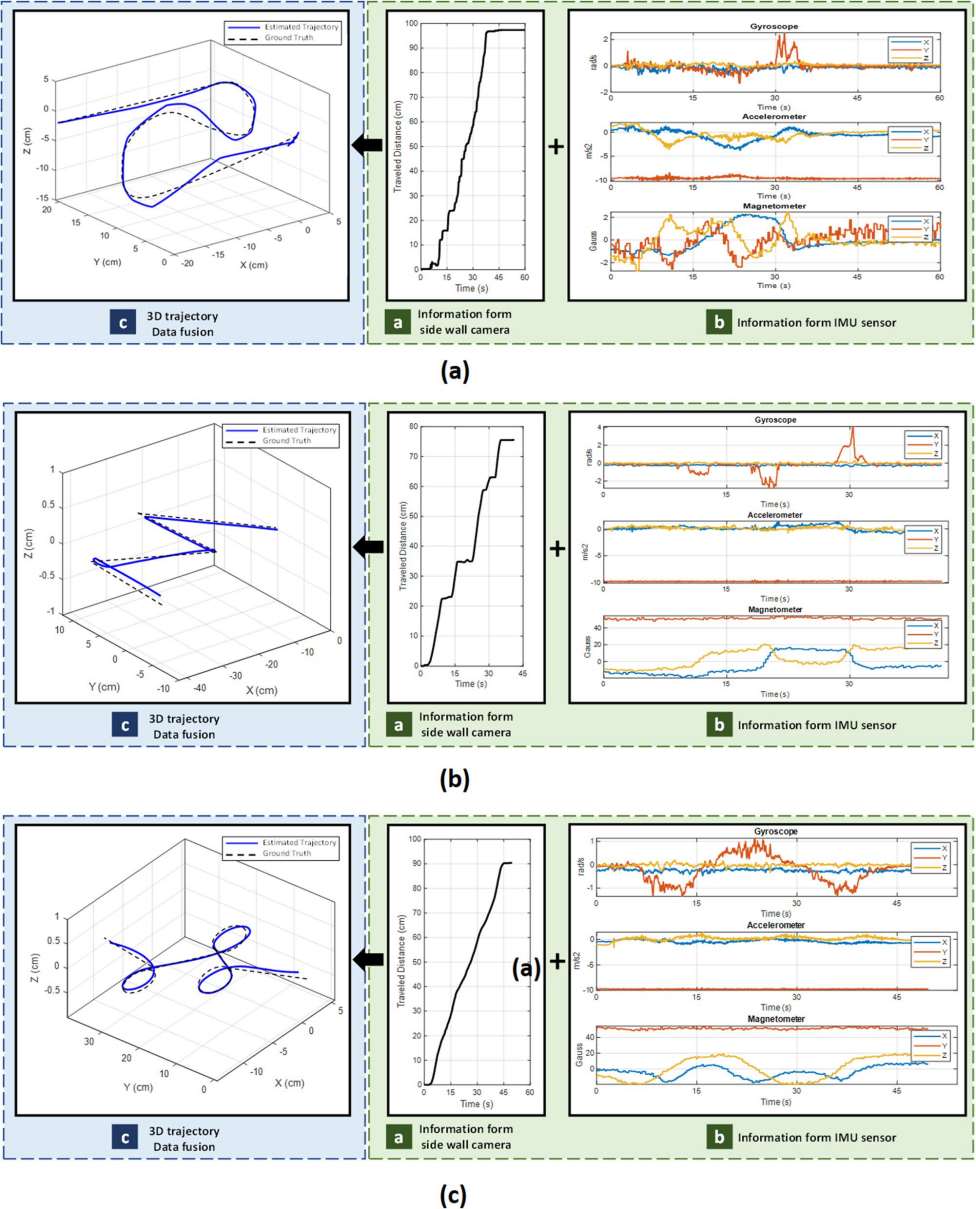
3D tracking for several trajectories. In each item, traveled distance, IMU data and computed trajectory are reported.

In Fig. 17 shows results of moving the capsule on different trajectories simulating random 3D motions inside the GI tract. At each experiment, three plot categories are reported, (a) Cumulative displacement data from side wall camera which denoted as traveled distance, (b) Raw data from IMU sensor, and (c) The 3D traveled trajectory which is given by fusion algorithm as described in Eq. (6). The 3D trajectory plot is the fused output of all sensors. In this plot, the dashed line shows the ground truth and blue line shows the estimated trajectory from the proposed algorithm. According to the results, the proposed method could estimate all curves paths correctly. As a result, the overall shape of the traveled path within the small bowel is correctly estimated since the shape and size of the trajectory matched with the reference track. One point to note is that the side wall cameras works best when the sensor surface touches the GI wall, like small bowel. In some early sections of the GI tract like esophagus and stomach, the side wall cameras may result in some errors. An article by Kalantar-Zadeh et al.^23^, analyzed the gas mixtures of different organs in GI, and showed that gas sensing could be a promising method to consider in the future. So, integrating an Oxygen molecule (O_2_) gas sensor to the capsule could make the current capsule prototype a full-length tracker of the entire GI tract, something that can be explored in the future.

### Validation experiment

**Figure 18:**
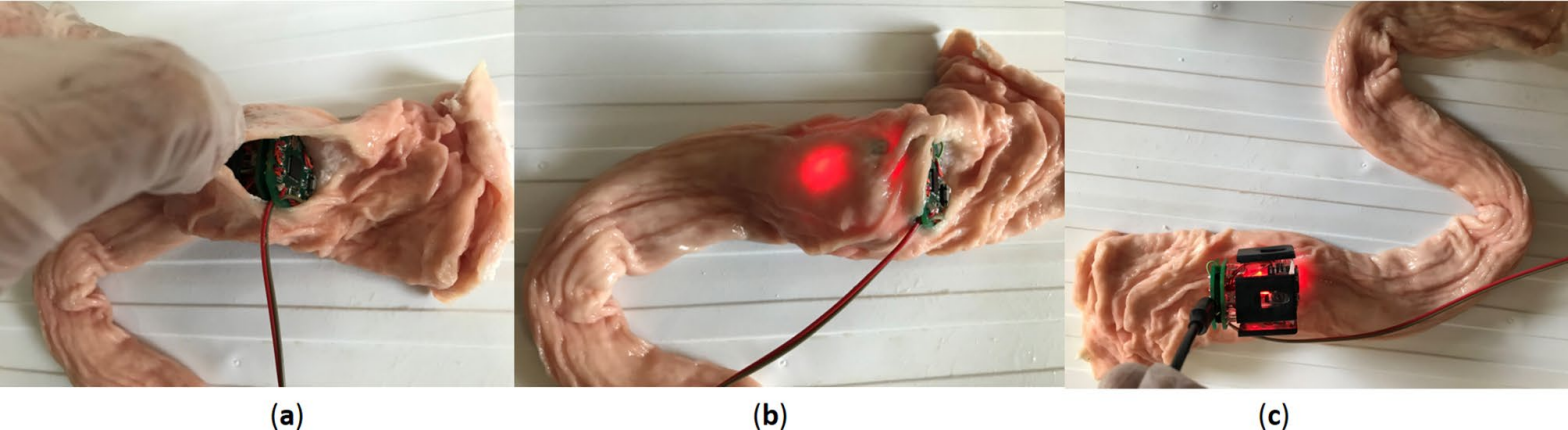
Test setup with porcine intestine.

The aim of this experiment is to examine the feasibility of tracking inside the intestine. Figure 18 shows the test setup for validation experiments. The capsule was manually inserted into the porcine intestine and pushed through it. Since it is an in-vitro experiment, the ground truth is measured by hand.

**Figure 19:**
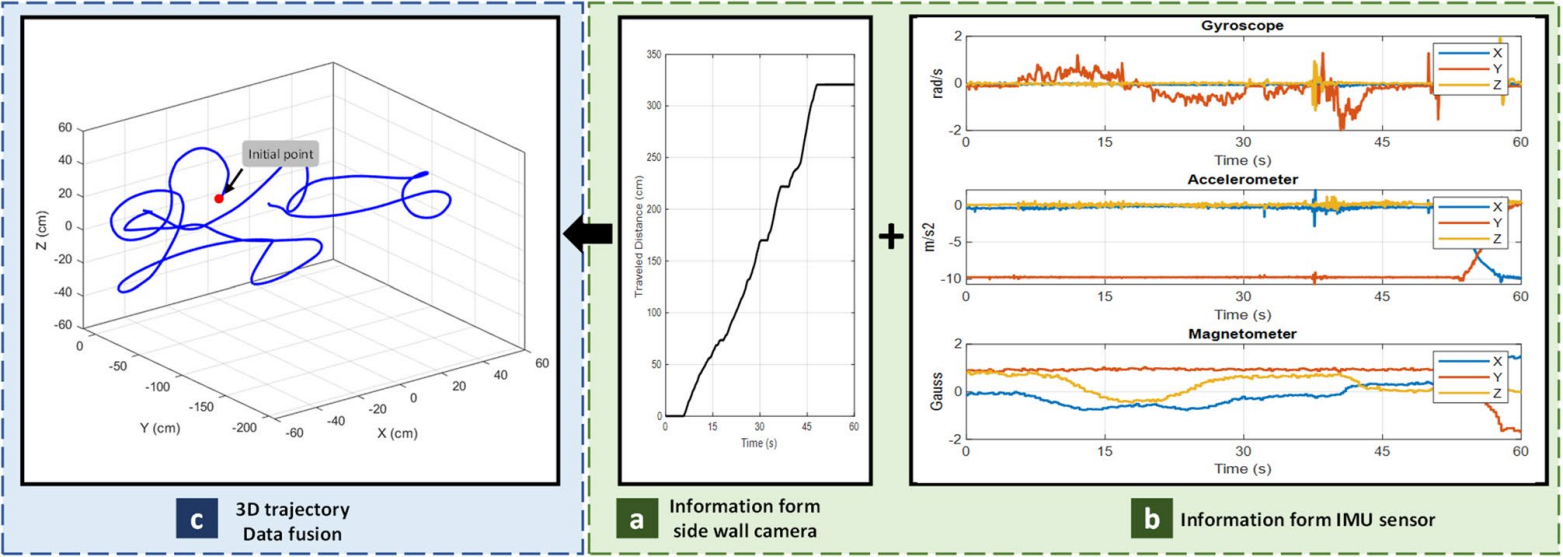
Validation experiments.

As depicted on Fig. 19, the proposed capsule and algorithm is able to perform localization inside in-vitro intestine.

The entire raw data for experiments can be found as Supplementary (see “Supplementary Information”).

### Power measurement

Due to the small size of capsule, there is limited space remained for batteries. The battery’s capacity is limited, and it must power up the device throughout the process which takes around 8 h. At this section, the power consumption of the capsule is investigated. Table 1 presents the operating voltage and current in active mode for the device at 3.3 V.

**Table 1:** Estimate of power consumption.

Component	Current (mA)	Power consumption (mW)
MPU9250	1.84	6.07
ATmega32	1.23	4.06
Red LED	1.28	4.22
Lora	5.25	17.32
Motion sensor	2.10	6.93
Total	11.70	38.61

In order to work properly for 8 h, the capsule needs battery with 94 mAh capacity. However, by integrating all the required components in an integrated chip (IC) or a system-on-a-chip (SoC) will lower the power consumption significantly than individual components.

Table 2 provides a list for several intra-body capsule localization methods and compares them based accuracy (how much the estimated measurement is close to the ground truth), extra hardware or weight inside capsule, patient’s comfort (i.e., patients’ mobility during the period of diagnosis), static reference point (patient’s outside movement vs capsule’s inside movement), interference of transmitted signal with other sources, involuntary GI motion, and prototype validation (in vitro or in vivo).

**Table 2:** Comparing available WCE localization methods.

Localization method	Accuracy (cm)	Additional hardware inside the capsule	Additional hardware for patients (patient’s comfort)	Static position for reference	Interference	Considering GI involuntary motion	In vitro or in vivo validation	Refs.
**RF**								
ToA, Received Signal Strength Indicator (RSSI)	0.2	NO – all necessary modules for RF localization is already implemented in capsules	Extra hardware (antenna array mounted on a cube) must carried by the patients. Limits the patient’s mobility significantly	YES—An array of antennas or a belt is required. Antennas must have fixed positions	Different tissues and muscles lead to inhomogeneous path loss and huge error in localization	Involuntary motion makes GI organs move inside body coordinate while the outside antenna cube is stationary. Results in large error in localization since the RF method is not able to distinguish between these two motions	NO	^24^
DoA	1						NO	^10^
ToA/RSS and spatial sparsity	0.8						NO	^25^
**Image-based**								
Hybrid video motion tracking & RF	2.3	NO—The frames captured by the capsule are used for localization	No interference with the patient's mobility	NO—There is no internal reference used to measure the accurate displacement	Fluctuation or inappropriate light. Poor quality imaging, low frames rate causes error in displacement measurement	Image-based localization is partially immune to peristalsis or involuntary motions, but over time, more errors from the missing frames are introduced	YES—The dataset from Pillcam	^23^
RCNN	3.5						YES—Invitro validation	^18^
**Ultrasound imaging**								
Ultrasonic and MRI	0.2	NO—The capsule is implemented with MRI and ultrasound friendly materials	Performed at hospital by laying on a bed. In addition, a doctor or technician is required to be present all times	YES—All distances are measured based on sensors’ position. Hence, it is considered as a fixed reference for localization	Ultrasound’s speed in different materials is the basis of displacement. But it varies in different organs and different human bodies which may cause errors	The GI organs are visible using this method; hence, we could compensate for the peristalsis motion	NO	^26^
**Radiation imaging**								
MRI compatible	0.3	NO—The capsule is implemented with MRI friendly materials. Capsule fabrication is expensive	Performed at hospital by laying on a bed. In addition, a doctor or technician is required to be present all times. Risk of exposing to radiation	YES—The position is measured in coordinates of the MRI device	The electronic devices are not allowed because they cause problems with MRI devices and introduce noises. High level of radiation	The GI organs are visible using this method; hence, we could compensate for the peristalsis motion	NO	^27^
**Magnetic**								
On-board magnetic sensing	0.5	YES—A small magnet is installed inside the capsule	An array of hall sensors is attached to the body which limits the patient’s mobility significantly	YES—An array of hall sensors is required. Antennas must have fixed positions	Interference with other sources of magnets. Such as ferromagnetic materials, wires with high current, etc	Involuntary motion of the GI system inside the body leads to loss of the track of the capsule. Similar issues like RF methods	NO	^28^
Jacobian-based iterative algorithm	0.7						NO	^29^
Magnetic sensing	0.5						YES—In vivo validation on a porcine	^30^
**Proposed method**								
IMU sensor and sidewall cameras	0.95	YES—Sidewall camera and IMU sensor	No additional hardware required. No interference with the patient's mobility	NO—Track from beginning to the end of GI path with no fixed reference point	External magnetic field may affect the IMU sensor	Involuntary and peristalsis motions have no interference with the actual motion	YES—In vitro validation on porcine intestine	

The upside of RF and magnetic-based localization is high accuracy, and they provide a global positioning information. However, these methods require a static reference antenna surround the patient body such as a box which interrupt person’s daily activities and suffer from external interferences. Radiation and ultrasound-based methods are not feasible as they require hospital facilities and expose patients to unwanted radiation. Image-based tracking methods alone are not reliable for complete localization, due to the lack of fixed marker inside GI and image blurriness. Furthermore, all the above methods fail to consider involuntary motion of small bowel which leads to errors in localization.

The proposed method has several significant advantages. Hospital facility and technician are not required in this method, and it does not confine patient’s movement. Most importantly, both patient’s external movement and the small bowel’s involuntary movement will not interfere with the capsule’s internal movement, and the actual displacement of the capsule can be projected. This is because the involuntary motion is rejected by the side wall cameras which measure the displacement while the capsule is moving inside the intestine and eliminate the GI motion inside the body.

As described on introduction, accuracy alone is not a reliable factor for comparing different methods. Instead, there are several factors contributes for selecting a method, such as considering GI involuntary motion, patient’s comfort, need for static external reference point, hospital facility and etc. Finally, the proposed method is experimentally validated in-vitro using multiple motions. However, there are still rooms for improvement and future research is directed towards further improving the WCE localization by improving the sensors and integrating more sensors like, pH and gas sensors for sections like stomach.

## Conclusion

In this paper we have developed a new method for WCE localization by incorporating an IMU sensor and four side wall cameras. The IMU sensor has gyroscope, accelerometer and magnetometer. The data from IMU sensors are fused together with the aim of fusion algorithm to estimate the orientation of the capsule. The side wall cameras are similar to an optical mouse sensor which has 18 × 18 pixels CCD and image processing part in a same chipset. By utilizing a four-camera topology ensures that at least one of them will be in constant touch of the GI wall, and measure the true displacement. Finally, the fusion algorithm combines both orientation and displacement to generate the 3D trajectory. The proposed device has no external reference point. Therefore, comparing with other capsule localization methods this system has many robust features including no interference due to patient’s external voluntary movement and small bowel’s involuntary internal movements.

## Supplementary Information

Supplementary Information.
